# Yigu decoction regulates plasma miRNA in postmenopausal osteoporosis patients: a randomized controlled trial

**DOI:** 10.3389/fphar.2024.1460906

**Published:** 2024-11-06

**Authors:** Haifeng Chen, Ruikun Zhang, Guijin Li, Kun Yan, Ziqi Wu, Yang Zhang, Zhineng Chen, Xinmiao Yao

**Affiliations:** ^1^ The Third Clinical Medical College of Zhejiang Chinese Medical University, Zhejiang Chinese Medical University, Hangzhou, Zhejiang, China; ^2^ Department of Orthopedics, The Third Affiliated Hospital of Zhejiang Chinese Medical University, Hangzhou, China

**Keywords:** yigu decoction, postmenopausal osteoporosis, randomised controlled trial, plasma miRNA, miR-133a-3p

## Abstract

**Background:**

Postmenopausal osteoporosis (PMOP) is a serious condition that affects elderly individuals. Our previous study revealed that Yigu decoction (YGD) effectively improved bone mineral density (BMD) in elderly individuals, but the mechanism underlying this effect remains unclear. In this study, we investigated the relationships among YGD, microRNAs (miRNAs), and bone metabolism by assessing the effects of YGD on the miRNA levels in patient plasma to provide a scientific basis for treating PMOP with YGD.

**Methods:**

In this clinical trial, 60 patients were randomly assigned to the YGD group or the control group (ratio of 1:1) and treated for 3 months. The primary outcome measure was BMD, and the secondary outcome measures included plasma miRNA levels, visual analogue scale (VAS) scores, alkaline phosphatase (ALP) levels, anti-tartrate acid phosphatase (TRACP-5b) levels and traditional Chinese medicine (TCM) syndrome scores. We assessed the regulatory roles of miRNAs in PMOP patients by analysing publicly available data from the Gene Expression Omnibus (GEO) database. Bioinformatics methods were also used to explore the mechanism by which YGD regulates miRNAs that are involved in bone metabolism.

**Results:**

Compared with those before treatment, the BMD, ALP levels, TRACP-5b levels, TCM syndrome scores and VAS scores improved in both groups after 3 months of treatment (P < 0.05). A total of 82 miRNAs differed between the groups. After analysing data from the GEO database, we confirmed that miR-133a-3p is the key molecule that mediates the effects of YGD intervention on PMOP. GO analysis of key genes suggested that gene enrichment was more pronounced in response to hormones, cellular response to growth factor stimulation, and positive regulation of physiological and metabolic processes. KEGG analysis revealed that these genes were enriched mainly in the PI3K-Akt, FOXO, and JAK-STAT pathways and other pathways. The results of the protein‒protein interaction (PPI) network analysis revealed that epidermal growth factor receptor (EGFR), Insulin-like growth factor 1 (IGF-1), Caveolin-1 (Cav-1) and others were core proteins.

**Conclusion:**

This study demonstrated that YGD is beneficial in the treatment of PMOP, ameliorating clinical symptoms and bone turnover indices. Moreover, the inhibition of miR-133a-3p expression may be the key mechanisms by which YGD regulates bone metabolism in the treatment of PMOP, although YGD regulates bone metabolism in a multitarget and multipathway manner.

## 1 Introduction

Postmenopausal osteoporosis (PMOP) is a common bone metabolism disease that affects middle-aged and elderly women, and it has emerged as a significant public health concern worldwide (Sozen et al., 2017). The pathogenesis of PMOP is primarily attributed to a rapid decrease in the levels of estrogen, or even a lack of estrogen, in the female body; this results in increased bone resorption by osteoclasts, which in turn accelerates bone loss and increases bone fragility, thereby significantly increasing the risk of fracture (Khosla et al., 2012). By 2035, the direct healthcare costs incurred in the management of osteoporotic fractures in China are projected to increase to approximately $19.92 billion, and costs associated with female patients are projected to account for approximately $16 billion, which is nearly fourfold higher than the costs projected to be associated with male patients (Si et al., 2015). Clinically, PMOP is treated mainly with drugs that are designed to inhibit bone resorption, such as bisphosphonates, selective estrogen receptor modulators, and Denosumab (Compston et al., 2017). Although these drugs can alleviate bone loss, their long-term use is associated with side effects, and there is a high risk of the development of drug resistance (Cauley, 2013; Gregson and Compston, 2022). These findings call for further investigations into the intrinsic mechanisms underlying bone metabolism, and such investigations will provide ideas for the development of novel therapeutic strategies.

In China, traditional Chinese medicine (TCM) is widely used in the treatment and prevention of PMOP. Studies have demonstrated that TCM is cost-effective, safe and efficacious (Chen et al., 2022). In addition, numerous herbal formulations have been shown to have good clinical effects on PMOP, as shown by their ability to regulate bone metabolism and increase bone mineral density (BMD) (Zhuo et al., 2022; Wang et al., 2024; Cao et al., 2024). As a component of TCM, Yigu decoction (YGD) is composed of several medicinal herbs, including Fructus psoraleae, Rhizoma Drynariae, Radix Rehmanniae, Salvia miltiorrhiza, Epimedii Folium and Dioscorea opposita. These herbs can promote osteogenesis, inhibit bone resorption, regulate calcium and phosphorus metabolism, and mitigate oxidative stress. With respect to the treatment of osteoporosis (OP), animal models have shown that some components of YGD, including icariin, naringin, and psoralen, have significant benefits (Duan et al., 2023). Our previous research revealed that YGD significantly alleviated pain and increased bone metabolism in OP patients (Y et al., 2015; Zhang et al., 2023). Additionally, we used an animal model of OP to evaluate the effects of YGD and examine its impact on bone metabolism-related signalling pathways (L et al., 2016; Lin et al., 2018; Yan et al., 2022a). Furthermore, proteomic analysis revealed that YGD treatment ameliorated OP by modulating the expression of proteins involved in bone tissue (Zhang et al., 2021). Through network pharmacology, we identified multiple signalling pathways that are involved in OP and are regulated by YGD, but the underlying mechanisms are not fully understood (Chen et al., 2021). Therefore, further research is needed to elucidate the mechanism of action of YGD in the treatment of PMOP.

MicroRNAs (miRNAs) are a small class of noncoding endogenous RNA molecules (approximately 19–25 nucleotides in length) that regulate the transcription and expression of target genes by degrading specific sequences or inhibiting translation (Lu and Rothenberg, 2018). miRNAs have been implicated in multiple biological processes, including bone metabolism (Tufekci et al., 2014). A recent study revealed that miR-18a-5p promotes the osteogenic differentiation of mesenchymal stem cells by downregulating Notch2 expression, suggesting that these molecules are potential therapeutic targets for the treatment of PMOP (He et al., 2024). Gu et al. demonstrated that mechanical stress can promote osteoblast differentiation and ameliorate OP via the miR-187–3p/CNR2 signalling pathway (Zhang et al., 2024). Collectively, these findings indicate that further investigations of miRNAs are needed to explore their roles in PMOP. In recent years, significant research on the role of TCM in controlling OP has focused on miRNAs. For example, Teng et al. reported that icariin may promote the osteogenic differentiation of bone marrow mesenchymal stem cells by upregulating miR-335–5p expression (Teng et al., 2022). Another study revealed that Xian-Ling-Gu-Bao enhances osteogenic differentiation by regulating the expression of miR-100–5p and its downstream pathways (Ai et al., 2021). These findings indicate that miRNAs hold great promise in the treatment of OP and may mediate the therapeutic effects of TCM.

This study aimed to explore the mechanism by which YGD modulates bone metabolism in patients with PMOP and the roles of miRNAs in this process. PMOP patients were treated with YGD for 3 months; the results indicated that the clinical symptoms and bone resorption indices of the patients improved, and 82 differentially expressed miRNAs were identified. Moreover, an analysis of data from the Gene Expression Omnibus (GEO) database identified miRNAs that participate in PMOP. Additionally, bioinformatics analyses were performed to predict the core targets of key miRNAs that are involved in the treatment of PMOP. This is the first study to investigate the epigenetic effects of YGD in patients with PMOP. Our results provide evidence for the clinical efficacy of YGD in treating PMOP. The results also provide new insights into the pathogenesis and treatment of PMOP, with a focus on the roles of miRNAs.

## 2 Methods

### 2.1 Study design

**FIGURE 1:**
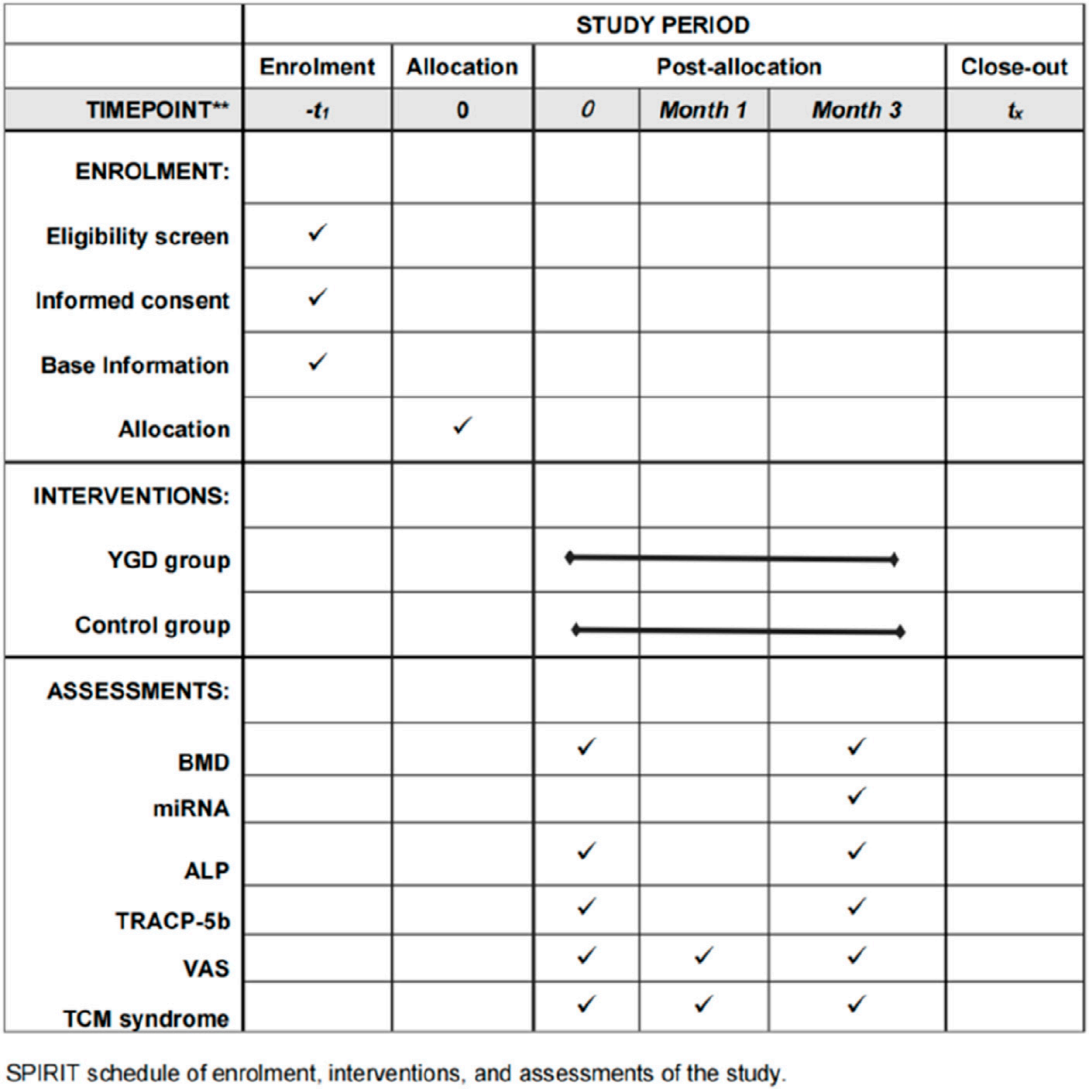
SPIRIT schedule of the trial. *Note: BMD: Bone Mineral Density miRNA: MicroRNA ALP: alkaline phosphatase TAACP-5b: tartrate acid phosphatase VAS: Visual Analogue Scale TCM Syndrome Score: Traditional Chinese medicine Syndrome Score*.

This study employed a single-blind, randomized controlled trial design. Participants were rigorously selected based on established diagnostic, inclusion, and exclusion criteria. After enrollment, participants were randomly assigned to groups, and the trial proceeded until the desired number of cases was observed. The recruitment period is from September 2022 to June 2024. The trial population consisted of PMOP patients from the Third Clinical College Hospital of Zhejiang Chinese Medical University. Eligible patients were randomly assigned (1:1) to either the YGD group or the control group. The report adhered to the SPIRIT checklist (Chan et al., 2013; Hoffmann et al., 2014). The SPIRIT schedule for the trial was shown in Figure 1. All included patients signed an informed consent form.

### 2.2 Eligibility criteria

#### 2.2.1 Inclusion criteria

(1) Diagnostic criteria and age criteria

The diagnosis is based on the “Recommended Diagnostic Standards for Osteoporosis in Chinese People (Third Draft)” developed by the World Health Organization (WHO) and the Osteoporosis Diagnostic Standards Discipline Group of the Chinese Gerontology Society Osteoporosis Committee (Zhang et al., 2014). Dualenergy X-ray absorptiometry (DXA) reports BMD as a T-value, reflecting the ratio of the current measurement to peak bone mass. OP is defined as a T score ≤ −2.5, based on the T score derived from DXA (de Villiers, 2024). The T-value of the lumbar spine served as an inclusion criterion for this trial.

Age criteria: postmenopausal women aged 45–75, with a natural menopause of more than 1 year (Weiyi et al., 2022).

(2) Those who have not taken anti-osteoporosis drugs and other treatments within 3 months as inclusion criteria.
(3) Patients who are willing to take herbal tonics for treatment.
(4) No bad habits (Long-term consumption of strong tea and coffee, alcoholism and vegetarianism), independent mobility.
(5) Sign the informed consent form for the clinical trial.

#### 2.2.2 Exclusion criteria

(1) Patients with senile osteoporosis and secondary osteoporosis (Table 1).
(2) Patients with fresh fractures.
(3) Patients who have taken TCM treatment within the last month.
(4) Patients with combined serious chronic primary diseases such as cardiovascular, cerebrovascular, hepatic, renal, respiratory and hematopoietic system and various digestive system diseases, and psychiatric patients.

**TABLE 1:** Patients with secondary osteoporosis.

Classification of secondary osteoporosis
1. Endocrine cortisolism, hyperthyroidism, primary hyperparathyroidism, acromegaly, hypogonadism, diabetes mellitus *etc.* 2. Nutritional protein deficiency, vitamin C deficiency, low calcium diet, alcoholism *etc.* 3. Hereditary osteogenesis imperfecta chromosomal abnormalities *etc.* 4. Renal Disease chronic nephritis hemodialysis *etc.* 5. Liver disease 6. Drugs corticosteroids, antiepileptics, antineoplastic drugs (such as methotrexate), heparin *etc.* 7. Other causes of osteoporosis or reduced bone mass

### 2.3 Intervention

#### 2.3.1 Control group

Element calcium 600 mg/day: Calcium Carbonate Chewable Tablets (100 mg/tablet, Hisun Pharmaceutical, Zhejiang, China) twice daily, 3 tablets each time. Vitamin D 800 IU/day: Vitamin D Drops (400 IU/capsule, Sinopharm Star Shark, Fujian, China) twice daily, 1 capsule each time. Alendronate sodium tablets 70 mg/week: Alendronate Sodium Tablets (70 mg/tablet, Merck Hangzhou, Hangzhou, China) once a week.

#### 2.3.2 YGD group

Element calcium 600 mg/day, vitamin D 800 IU/day, alendronate sodium tablets 70 mg/week + YGD (Dioscorea opposite 15 g, Radix Rehmanniae 15 g, Rhizoma Drynariae 15 g, Fructus psoraleae 10 g, Salvia miltiorrhiza 30 g, Epimedii Folium 15 g as granules) The medication, provided by the Third Clinical College Hospital of Zhejiang Chinese Medical University, should be prepared by dissolving it in 200 mL of warm boiled water. The dosage is one cup in the morning and one cup in the evening, to be taken continuously for a period of 3 months.

### 2.4 Outcomes

#### 2.4.1 Primary outcomes

DXA bone densitometry (GE Healthcare, United States) was employed to measure changes in lumbar BMD in patients following a 3-month treatment period, with the aim of evaluating treatment efficacy.

#### 2.4.2 Secondary outcomes

TCM Syndrome Score: The scoring was done according to the TCM standard mapping TCM evidence scale promulgated by the Chinese Medicine Industry Association of the People’s Republic of China. Refer to the Traditional Chinese Medicine Diagnosis and Treatment Guidelines for Postmenopausal Osteoporosis (Osteoporosis) (2019 Edition) (Chinese Society of Traditional Chinese Medicine, 2020). TCM syndrome scores were assessed for participants in both groups at baseline, and subsequently at one and 3 months post-treatment initiation. (Table 2).

**TABLE 2:** TCM symptom grading scale.

Symptoms	Standard for evaluation				Score
	0	1	2	3	
Low back pain^a^ (patient self-rated, VAS score)	Normal	Level 1–3	Level 4–6	Level 7–10	
soreness and weakness of waist and knees^b^	Normal	After walking (≥1000 m)	After walking (≥300 m,≤1000 m)	After standing or walking (<300 m)	
Lower limb cramps^b^	Normal	≤2 times per month	2–10 times per month	≥10 times per month	
trudge^c^	Normal	Walking ≥100 m	Walking ≥10 m,≤100 m	Walking ≤10 m Or unable to stand	
weight loading ability^c^	Normal	feeling of powerlessness	feeling the pinch	unweightable	

*Note: Calculate Score Class*.

^a^ × 3; Class.

^b^ × 2; Class.

^c^ × 1.

Bone metabolism indicators: All specimens were collected between 6:00 a.m. and 8:00 a.m., both prior to treatment and 3 months post-treatment, via 4 mL venipuncture from the elbow vein. Following collection, serum samples were immediately centrifuged, and the serum was subsequently harvested. Enzyme-linked immunosorbent assay (ELISA) was performed to quantify serum levels of alkaline phosphatase (ALP) and tartrate-resistant acid phosphatase (TRACP-5b) using the Human ALP ELISA Kit (ELK 1941) and Human TRACP-5b ELISA Kit (ELK9590) from ELK Biotechnology (Wuhan, China), according to the manufacturer’s protocol.

Visual Analogue Scale (VAS): A 10-cm horizontal line, with endpoints marked at 0 for no pain and 10 for severe pain, was drawn on the paper to depict varying levels of discomfort. Participants were instructed to indicate their level of pain by marking a line segment corresponding to their perceived discomfort. VAS assessments were conducted for participants in both groups at baseline, and subsequently at one and 3 months post-treatment initiation.

### 2.5 miRNA and bioinformatics

miRNA: All specimens were collected between 6:00 a.m. and 8:00 a.m. Additionally, 4 mL of venous blood from the elbow was collected 3 months post-treatment. The extracted plasma was stored at a temperature of −70°C for preservation and transported on dry ice to maintain its integrity. Initial assessments were performed to verify the quality of the samples. High-throughput sequencing platforms were utilized to sequence miRNA fragments. In this experiment, a library was constructed using the Illumina TruSeq Small RNA Kit, and the obtained sequences were subjected to quality control before being compared against the miRBase and Rfam databases for miRNA identification (Kozomara et al., 2019; Kalvari et al., 2021).

The GEO database is a publicly accessible repository that archives gene expression data, including profiles of gene expression, transcriptomes, and miRNA. The database was utilized to identify miRNAs potentially associated with PMOP to facilitate further investigation into the underlying mechanisms. We used 8 databases including DIANA-microT, (Maragkakis et al., 2009), ElMMo, (Gaidatzis et al., 2007), MicroCosm, (He et al., 2018), miRanda, (Liu et al., 2019), miRDB, (Sun et al., 2019), PicTar, (Lu et al., 2017), PITA, (Gu et al., 2020), and TargetScan (Zhu et al., 2023) to predict the targeted genes of miRNA. We used the GeneCards database to predict genes related to PMOP (Stelzer et al., 2016). Subsequently, we identified common targets associated with PMOP and miRNA, and analyzed these targets using the Gene Ontology (GO) and the Kyoto Encyclopedia of Genes and Genomes (KEGG) databases (Chen et al., 2017). According to the STRING database, we used Cytoscape (v3.9.0) software to construct a Protein-Protein Interaction (PPI) network diagram for proteins.

### 2.6 Data collection and follow-up

Demographics and Baseline Analysis: We collected demographic information (participant age, height, *etc.*) and baseline characteristics (disease history, medication history, *etc.*). Participant received baseline and follow-up visits at 4 and 12 weeks at the Third Clinical College Hospital of Zhejiang Chinese Medical University. Data collection was performed by independent researchers who underwent training before the commencement of the trial.

### 2.7 Sample size calculation

The BMD of participants is the primary outcome indicator. Based on the results of previous small sample experiments and previous studies, it is assumed that the difference between the experimental group and the control group is 0.2, and the standard deviation is 0.26 (Zhang et al., 2023). With a two-side significance level of α = 0.05 and power of 80%, we obtained a minimum sample size of 27 cases per group. Considering the dropout rate of 15%–20%, 30 samples will be required per group for a total of 60 cases.

### 2.8 Randomization, blinding, and treatment allocation

The allocation sequence was generated by a statistician who was not involved in the clinical trial. Using SAS statistical software, the statistician generated a total of 60 tables of random numbers in a 1:1 ratio for the YGD and control groups, which were kept in brown, opaque, sequentially numbered envelopes. The envelopes were opened by the researcher after participants who met the criteria had signed the informed consent form. The procedure was kept confidential for the participants and the statistician. Considering the possible adverse effects of the drug, we adopted a single-blind design (subjects). To minimize bias from single-blinding, data collection was performed by a third party not involved in the trial. Subjects were unaware of whether they are in the experimental or placebo group, while ensuring that there is no difference in appearance between YGD and placebo. Placebo was manufactured by Huisong Pharmaceuticals. Placebo is physically similar to YGD in size, taste, odor and color and consists of corn starch, pre-gelatinized starch, maltose, caramel coloring and water. Since the investigators are not blinded, we do not anticipate that any unblinding will be required, but will report any unblinding if needed.

### 2.9 Data management

The investigator is required to fill in the case report form with the data collected according to the trial protocol and use EpiData 2.0 software to collect or record the data. The data management is the responsibility of a dedicated person, who must ensure the authenticity, completeness and accuracy of the clinical study data. At the end of the trial, the investigator will submit case report forms for all patients enrolled in this trial to the data management center, and these case report forms should be complete and signed.

### 2.10 Data analysis

All statistics were done by a third party not involved in the trial. The data analysis was conducted using SPSS software. For all statistical analyses, statistically significant at P < 0.05. The measurement data was expressed as mean ± standard deviation (‾x ± s) and the count data as percentage (%). When making comparisons between groups, a t-test for two independent samples is used if the normality test and chi-square test are satisfied. If the normality test is not satisfied, the Wilcoxon Man-Whitney U rank sum test will be used to compare the two samples. If the normality test is satisfied but not the chi-square test, two independent sample-corrected t-tests are used. When performing correlation analysis, Pearson correlation analysis will be used if the data meet normality, otherwise Spearson correlation analysis will be used.

### 2.11 Rejection, dropout criteria and adverse events

(1) Poor compliance, unwillingness to take herbal tonics, failure to follow the prescribed treatment or incomplete observation data that affect the assessment.
(2) Those who are treated with other treatments or other medications midway.
(3) Subjects who do not meet the inclusion criteria and are mistakenly included. Those who stop due to adverse reactions are not included in the efficacy analysis.
(4) Subjects who develop serious complications or other serious illnesses during the trial that require emergency measures.
(5) Subjects who have other reasons for not being able to continue this trial.

Emergency treatment: If during the process the patient’s disease condition worsens or cannot be relieved by the existing treatment, which seriously affects daily work and life, the emergency adjustment of the treatment plan may be used after evaluation and diagnosis by an orthopedic surgeon, and the specialist will assess whether to discontinue the trial. The date and time of use and dosage of each type of emergency medication or other therapeutic measure must be recorded in a timely manner.

Adverse Events: All adverse events that occurred during the trial were meticulously documented, including details of symptoms, severity, timing of onset, duration, and the management strategies employed.

## 3 Results

**FIGURE 2:**
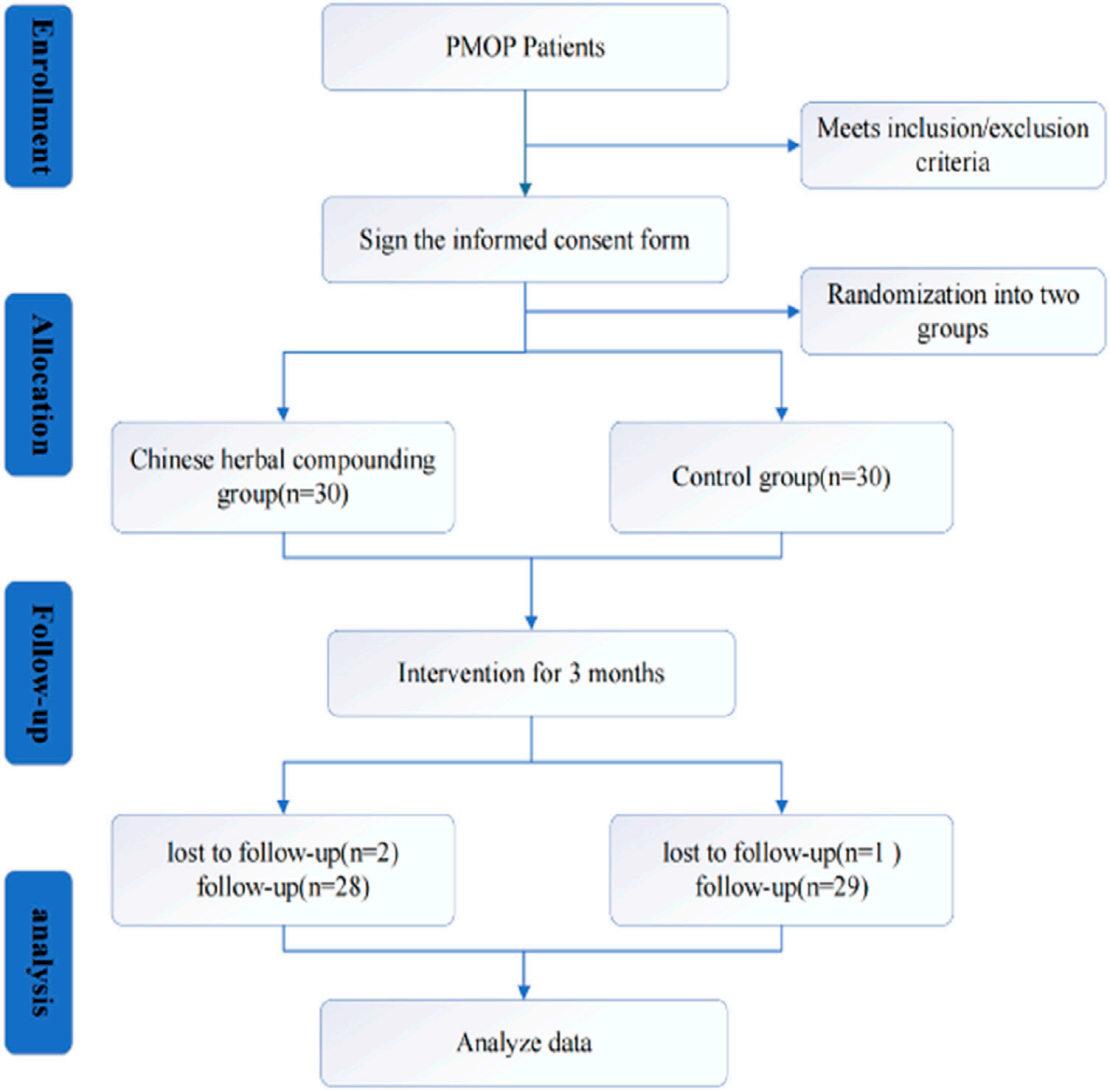
Study flow diagram.

A total of 60 patients were enrolled and randomly assigned to either the herbal group (n = 30) or the control group (n = 30). Of the 60 enrolled, 57 patients completed the study; one in the YGD discontinued due to gastrointestinal discomfort, and another was excluded for irregular medication adherence. One participant in the control group was excluded due to lost follow-up. The study flow is depicted in Figure 2.

### 3.1 Demographic characteristics of participants

As indicated in Table 3, no significant differences were observed in the demographic characteristics between the two patient groups. The YGD group patients had a mean age of (66.39 ± 3.69) years, weight of (55.18 ± 5.84) Kg, and height of (157.64 ± 5.30) cm, whereas the control group had a mean age of (65.24 ± 4.67) years, weight of (54.48 ± 6.31) Kg, and height of (157.93 ± 4.87) cm, with no statistically significant differences observed.

**TABLE 3:** Demographic characteristics of the two groups.

	N	Age	Height (cm)	Weight (Kg)
YGD group	28	66.39 ± 3.69	157.64 ± 5.30	55.18 ± 5.84
Control group	29	65.24 ± 4.67	157.93 ± 4.87	54.48 ± 6.31
*p*-value		0.461	0.737	0.424

### 3.2 Primary outcome

As shown in Table 4, the T- values of lumbar spine BMD of two groups were (−2.78 ± 0.22) and (−2.89 ± 0.26) for YGD and control group, respectively. There was no significant difference between the group (*P*> 0.05).

**TABLE 4:** Clinical indicators of two groups of patients.

		Control group	YGD group
pre-treatment	BMD	−2.93 ± 0.28	−2.84 ± 0.37
	ALP(U/L)	91.97 ± 9.03	92.43 ± 8.34
	TRACP-5b (U/L)	4.67 ± 0.67	4.70 ± 0.63
	VAS	5.03 ± 0.63	5.32 ± 0.67
	TCM symptom	16.62 ± 3.02	16.75 ± 2.50
After 1 month of treatment	VAS	1.48 ± 0.94^a^	1.25 ± 0.83^a^
	TCM symptom	11.76 ± 2.37^a^	11.32 ± 2.74^a^
After 3 months of treatment	BMD	−2.89 ± 0.26^a^	−2.78 ± 0.22^a^
	ALP(U/L)	84.38 ± 10.07^a^	88.39 ± 9.29^a^
	TRACP-5b (U/L)	3.50 ± 0.47^a^	2.98 ± 0.38^b^ ^,^ ^a^
	VAS	1.41 ± 0.93^a^	1.11 ± 0.60^b^ ^,^ ^a^
	TCM symptom	9.00 ± 2.49^a^	7.36 ± 1.83^b^ ^,^ ^a^

^a^ Compared to pre-treatment, *P*< 0.05.

^b^ Between-group comparison, *P*< 0.05.

### 3.3 Secondary outcomes

Following a 3-month treatment period, the YGD group exhibited superior improvement in the TRACP-5b index compared to the control group (P < 0.05), whereas no significant difference was observed in ALP levels (P > 0.05). Both groups experienced a significant decrease in TCM Syndrome Scores, with the YGD group demonstrating greater efficacy (P < 0.05). Additionally, the YGD group reported lower VAS scores than the control group (P < 0.05), indicating significant pain relief in PMOP patients. Details are displayed in Table 4.

### 3.4 Plasma miRNA and bioinformatics

**FIGURE 3:**
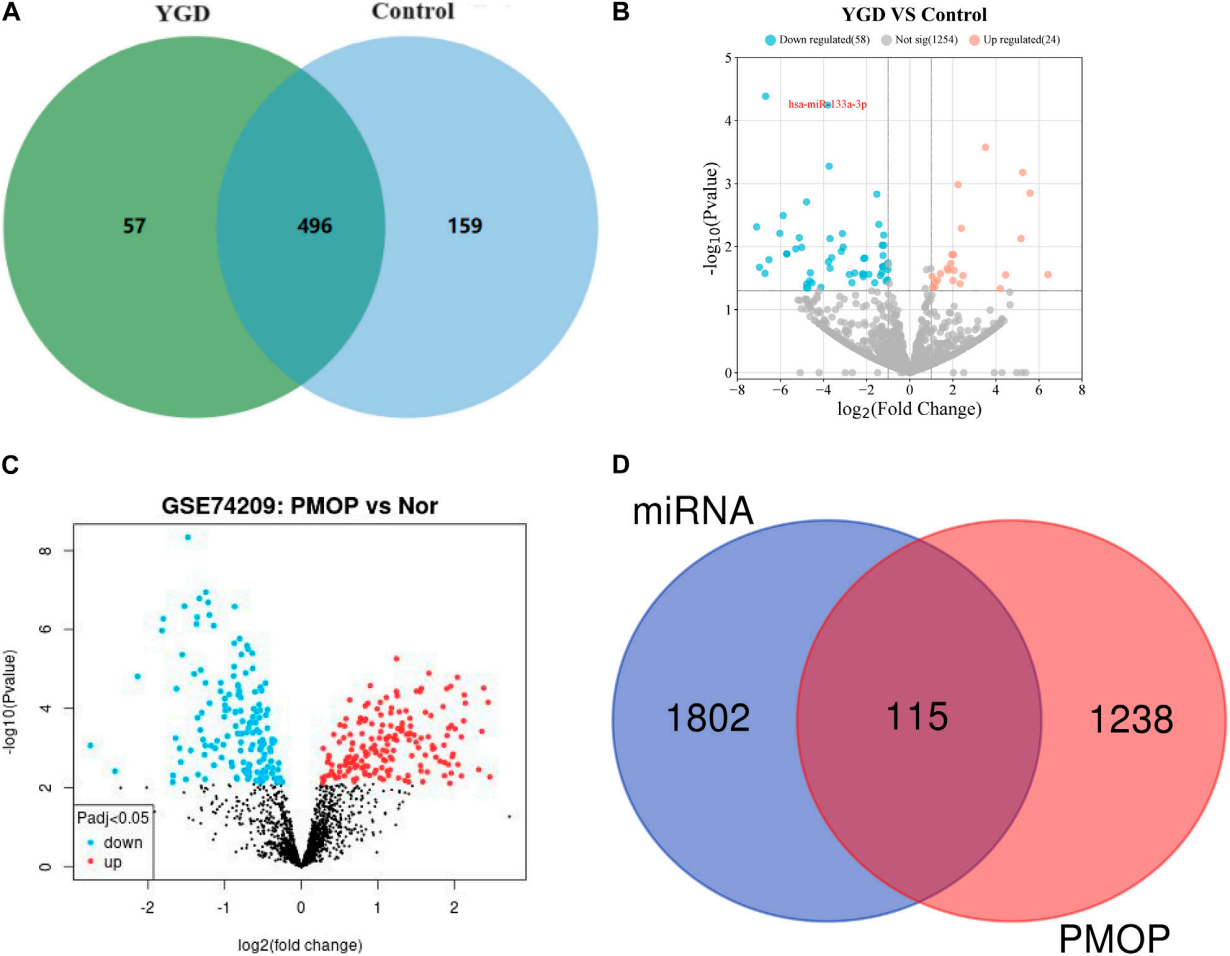
Differential expression of miRNAs. Note: **(A)** Venn plots of plasma miRNAs in two groups of patients. **(B)** Volcano plot of differential expression of miRNA between two groups of patients. The red circle represents upregulated miRNAs in the YGD group, the blue circle represents downregulated miRNAs, and the gray circle represents no significant difference in miRNA. **(C)** Volcano plot of miRNA in patients with GSE74209 data. The red dots represent upregulated miRNAs in the OP group, the blue dots represent downregulated miRNAs, and the black dots represent undifferentiated miRNAs. **(D)** Venn diagram of miRNA targets and PMOP pathogenic genes.

Using high-throughput sequencing, detect the differences in plasma miRNAs between two groups of patients (5 randomly selected from each group) after 3 months of treatment. The Illumina sequencing platform was used to sequence small RNA (sRNA) from two groups of patients, and quality control was performed on the original sequencing data, such as removing adapter sequences, removing short sRNA (<18 nt), and removing long sRNA (>32 nt). Compare the processed sequences that match the length with miRBase and Rfam databases to determine the miRNA name and structure (Zhao et al., 2022). Use Venn diagrams to visually express shared and unique miRNAs between different groups. Among them, the treatment group had 553 types of miRNAs, the control group had 655 types of miRNAs, and a total of 496 types of miRNAs were present in both groups of patients. Details are displayed in Figure 3A (The data on detected miRNAs have been submitted as Supplementary Appendix SA1).

The expression levels of miRNAs from both patient groups were statistically analyzed, and the expression levels were normalized using scripts per million (TPM) (Zhao et al., 2020). DESeq2 software was utilized to analyze the differential expression of miRNAs between the two patient groups. The screening criteria included: P values <0.05, and |Log2 (fold change)| > 1, to identify differentially expressed genes. The results indicated that the expression of 82 miRNAs was significantly altered in the YGD group compared to the control group. Of the differentially expressed miRNAs, 24 were upregulated (Log2 fold change >1) and 58 were downregulated (Log2 fold change < −1). The volcano plots illustrate the significantly differentially expressed miRNAs between the two groups, as depicted in Figure 3B.

The GSE74209 dataset, sourced from the GEO database, was publicly available. The dataset comprises miRNA data from 12 female patients, 6 with PMOP and 6 with normal BMD. A total of 324 miRNAs were identified as differentially expressed between the groups (P < 0.05). GEO2R software was utilized to compare differentially expressed miRNAs between the two patient groups, and the results were visualized using a volcano plot, as depicted in Figure 3C.

Through comparison of the two datasets, several miRNAs, including miR-219a-5p, miR-133a-3p, miR-138–5p, miR-551a, and miR-5187–5p, were identified as potentially critical for the treatment of PMOP with YGD. miR-133a-3p was selected for further investigation due to its significant differential expression (P = 5.68E-05) and the well-documented role of miR-133a-3p in bone metabolism (Makitie et al., 2020; Pala and Denkceken, 2019; Wang et al., 2017).

To elucidate the function of miR-133a-3p, the multiMiR R package was utilized to predict its functional roles. The multiMiR package integrates predictions from eight miRNA function databases, including DIANA-microT, TargetScan, PicTar, miRanda, PITA, miRDB, ElMMo, and MicroCosm (Ru et al., 2014). A total of 2069 related genes for miR-133a-3p were identified. After removing duplicates, 1917 unique genes were predicted to be associated with miR-133a-3p (Data have been submitted as Supplementary Appendix SA2). To identify key genes targeted by miR-133a-3p in PMOP, a search was conducted for all 1,353 genes associated with PMOP using the GeneCards database (https://www.genecards.org/). A total of 115 key genes were identified by intersecting the 1,353 genes related to PMOP with the 1917 predicted target genes of miR-133a-3p. These 115 key genes are posited to form the core regulatory network of miR-133a-3p in the context of PMOP therapy. The Venn diagram is depicted in Figure 3D (Data have been submitted as Supplementary Appendix SA3).

**FIGURE 4:**
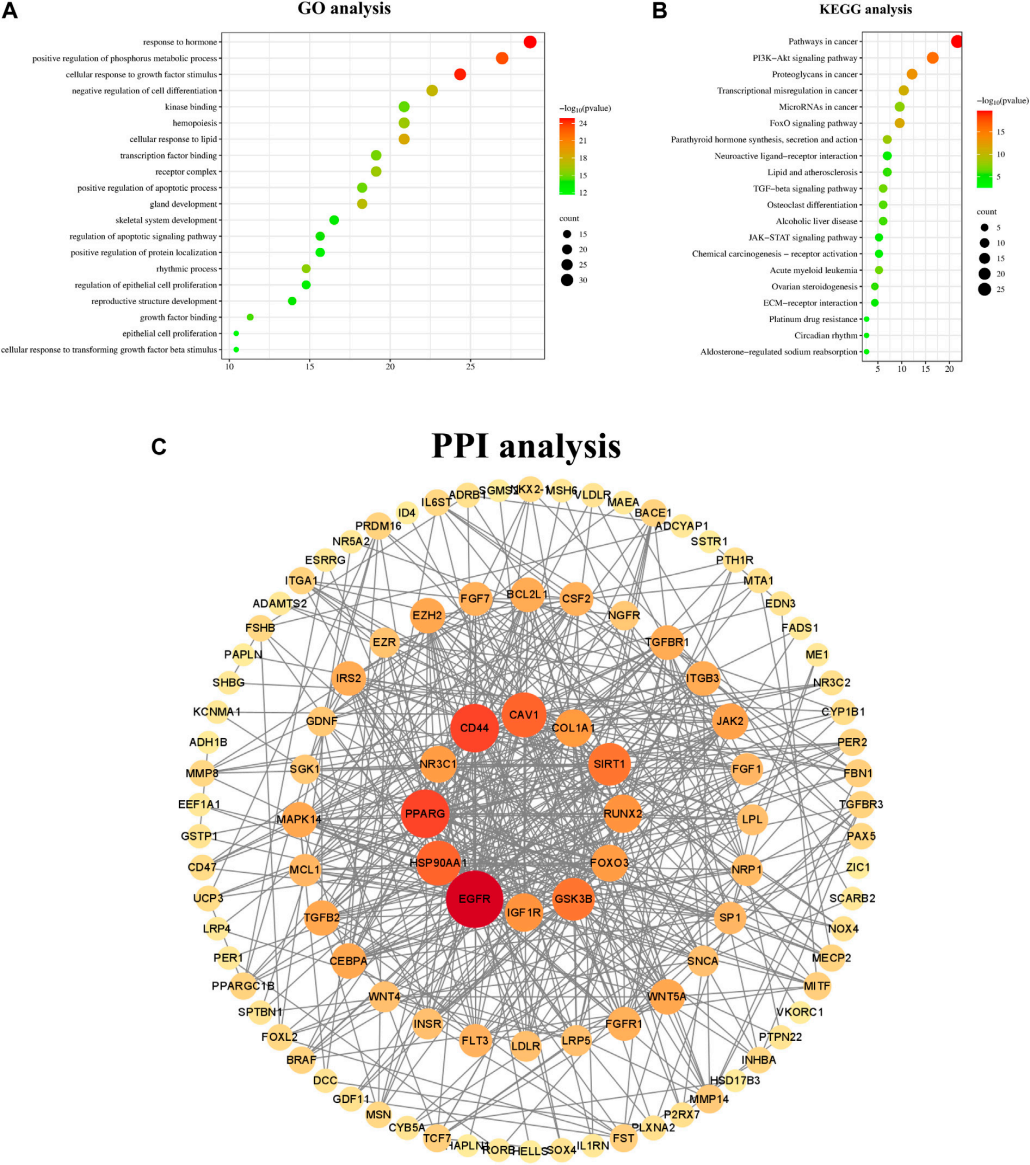
GO KEGG and PPI analysis of key genes. *Note:* **(A)** *GO analysis of key genes* **(B)** *KEGG analysis of key genes.* **(C)** *Protein-protein interaction network of common targets of PMOP and miR133a-3p. These circular nodes represent potential targets and the connecting lines represent the interactions between them; the larger the area of a node in the graph, the more important it is in the network.*

Additionally, KEGG and GO analyses were conducted to investigate the associated signaling pathways and functional annotations, with the findings presented in Figures 4A, B. The results of GO analysis indicated that gene enrichment is more pronounced in response to hormone, positive regulation of phosphorus metabolic processes, cellular response to growth factor stimulation, and positive regulation of physiological metabolic processes. The KEGG analysis revealed that these genes are predominantly associated with the PI3K-Akt signaling pathway, the FOXO signaling pathway, the JAK-STAT signaling pathway, and others. The STRING database was utilized for the analysis of PPI, and the results were visualized using Cytoscape 3.9.0 (Szklarczyk et al., 2023). Notably, EGFR was identified as the most central node, followed by IGF-1, Cav-1, Runx2, JAK, and others. The result is depicted in Figure 4C.

## 4 Discussion

This was a randomized, single-blind, placebo-controlled trial investigating the efficacy of YGD in the treatment of PMOP patients as well as the roles of miRNAs in the mechanisms underlying PMOP. The results revealed that YGD alleviated clinical symptoms, increased bone metabolism indices, and regulated the plasma levels of miRNAs in patients with PMOP. These findings indicate that YGD is an effective complementary therapy that can be used to treat PMOP patients.

Currently, numerous pharmaceutical drugs are used in the treatment of OP. However, these drugs do not comprehensively resolve the symptoms of patients, and their long-term efficacy has not been conclusively determined (Reid and Billington, 2022). TCM has been used to treat various diseases for thousands of years. As medical research has advanced, the application of TCM in the management of OP has attracted significant interest among researchers. Notably, YGD was found to effectively treat OP in basic research and clinical studies. A previous network pharmacology study revealed that YGD can ameliorate OP through multitarget and multipathway mechanisms (Chen et al., 2021). In rat models, YGD increases osteoblast proliferation and differentiation, decreases osteoclast activity and differentiation, and increases BMD (L et al., 2016; Lin et al., 2018; Yan et al., 2022a). A previous clinical trial revealed that YGD significantly prevents calcium loss, increases BMD, alleviates pain, and improves quality of life of patients with OP (Y et al., 2015; Zhengqing et al., 2016; Yan et al., 2022b). This study showed that YGD administration markedly ameliorates symptoms and VAS scores in PMOP patients compared with the control treatment (P < 0.05). These results demonstrated that YGD effectively alleviates clinical symptoms, including pain, in PMOP patients.

Previous studies have reported an annual decrease in BMD of approximately 0.5% in PMOP patients (Peacock et al., 2000). In this study, no significant difference in BMD was observed between the two groups after 3 months of treatment (P > 0.05), likely due to the short observation period. Given that BMD measurements have a margin of error between 1% and 5% and that changes in BMD occur gradually, a longer follow-up period is needed to fully evaluate the effects of YGD on BMD (van Schaick et al., 2015; Ravn et al., 1999). Second, alendronate treatment may affect BMD; thus, further research is needed to evaluate the independent effects of YGD. Bone turnover markers are often used to assess bone metabolic activity and changes in BMD (Nenonen et al., 2005). This approach has the advantages of better sensitivity and convenience than BMD. TRACP-5b is a key marker of bone resorption that is used assess the degree of bone resorption (Rui et al., 2024). Comparative analysis of the two groups revealed that the YGD group achieved greater improvement in TRACP-5b levels than did the control group (P < 0.05). These findings highlight the role of YGD in bone metabolism, particularly its effect on inhibiting bone resorption. Our findings are consistent with those obtained in our previous network pharmacology and animal model studies (Yan et al., 2022a; Chen et al., 2021). Nevertheless, the mechanisms by which YGD regulates bone resorption require further in-depth investigations.

In recent years, significant research has investigated the roles of miRNAs in the pathogenesis and progression of OP (Tufekci et al., 2014). miRNAs can regulate bone homeostasis and bone metabolism, and some miRNAs are considered important targets in OP. The results of this study indicated that YGD effectively regulated the miRNA levels in the plasma of patients with PMOP. Some of the identified miRNAs, such as miR-133a-3p, are closely related to OP. Multiple studies have demonstrated that the miR-133a-3p levels in the peripheral blood of PMOP patients are higher than those in normal subjects (Koutsoulidou et al., 2011). The upregulation of miR-133a-3p may reflect a decrease in BMD, suggesting that inhibiting the expression of miR-133a-3p might be an effective strategy for the treatment of PMOP. Studies have reported that miR-133a-3p regulates the key osteoclast transcription factor TRACP via the p38 (MAPK) pathway (Stephens et al., 2020). These findings are consistent with our findings, which revealed decreased miR-133a-3p levels in PMOP patients who were treated with YGD, which were accompanied by significant changes in TRACP-5b levels (P< 0.05). In addition, the results indicated that YGD treatment modulated the miRNA levels in the plasma of PMOP patients, with miR-133a-3p being a pivotal candidate that mediates the effect of YGD. Mechanistically, YGD may alleviate PMOP symptoms by downregulating miR-133a-3p expression levels, inhibiting bone resorption and modulating bone metabolism.

To elucidate the role of miR-133a-3p in the mechanism of action of YGD, bioinformatics analysis was conducted to identify potential target genes of miR-133a-3p. A total of 115 key genes were identified by intersecting the target genes of miR-133a-3p with the disease-associated genes of PMOP. KEGG pathway analysis revealed that the target genes were enriched in the PI3K-Akt signalling pathway. The regulatory role of miR-133a-3p in this pathway has been well documented. For example, studies on bone metastasis have shown that miR-133a-3p inhibits PI3K-Akt signalling by modulating various cytokines (Tang et al., 2018). The PI3K-Akt signalling pathway participates in the regulation of osteoblasts and osteoclasts (Liang et al., 2022). Therefore, we speculate that YGD may regulate bone metabolism by inhibiting miR-133a-3p expression and activating the PI3K‒Akt signalling pathway. The results of the PPI network analysis revealed that EGFR was the most highly enriched protein. These findings suggest that EGFR may be a crucial target by which YGD modulates miR-133a-3p in the treatment of PMOP. Evidence from previous investigations suggests that EGFR regulates osteoblast proliferation and differentiation, and osteoblast-specific EGFR-knockout mice present osteoporotic features, indicating that targeting EGFR may be an attractive strategy for treating PMOP (Linder et al., 2018). The EGFR signalling pathway regulates osteoclast function, affecting the differentiation and survival of osteoclasts via the RANK pathway (Yi et al., 2008). Furthermore, miR-133a-3p was found to be as an upstream regulator of EGFR (Xia and Jie, 2020). On the basis of this information, we believe that YGD might modulate bone metabolism via the miR-133a-3p/EGFR axis. Naturally, further rigorous experimentation is necessary to verify these speculations.

TCM has several advantages in the treatment of PMOP, including that it causes minimal side effects and is cost-effective. However, the mechanisms of action associated with herbal remedies are unclear, which has drawn criticism. Here, we found that YGD effectively ameliorated clinical symptoms and pain while enhancing bone metabolism, primarily by inhibiting bone resorption, in PMOP patients. YGD treatment altered the miRNA levels in the plasma of PMOP patients, suggesting that miRNAs participate in the mechanism by which YGD regulates bone metabolism. Nevertheless, this study has several limitations that should be acknowledged. First, the three-month treatment duration may be inadequate for capturing the complete bone metabolism cycle, and this short duration could mask differences in BMD and other relevant indicators. Therefore, future studies should extend the treatment and follow-up periods to better evaluate the long-term efficacy of YGD. Second, the small sample size and the selection of patients exclusively from one hospital may have introduced bias. Therefore, future studies with larger sample sizes from multiple centres are needed to address the present limitations. Additionally, the role of miR-133a-3p needs to be validated through well-designed experiments to elucidate the mechanism of action of YGD in PMOP.

This study demonstrated that after a three-month treatment period, YGD significantly improved clinical symptoms, pain, and bone metabolic indices, suggesting that YGD is a safe and efficacious complementary therapy for the treatment of PMOP. Mechanistically, YGD increased bone metabolism in patients with PMOP via a mechanism that involves miR-133a-3p and its downstream targets. Future large-sample, multicentre, double-blind, randomized controlled trials with extended treatment and follow-up periods are recommended to verify the long-term efficacy of YGD.

## Data Availability

The datasets presented in this study can be found in online repositories. The names of the repository/repositories and accession number(s) can be found in the article/Supplementary Material.
